# Expression and function of Kv1.3 channel in malignant T cells in Sézary syndrome

**DOI:** 10.18632/oncotarget.27122

**Published:** 2019-08-06

**Authors:** Tengpeng Hu, Terkild Brink Buus, Thorbjørn Krejsgaard, Anneline Nansen, Betina Kerstin Lundholt, Pieter Spee, Simon Fredholm, David Leander Petersen, Edda Blümel, Maria Gluud, Madalena N. Monteiro, Andreas Willerslev-Olsen, Mads Hald Andersen, Per thor Straten, Özcan Met, Veronica Stolearenco, Hanne Fogh, Robert Gniadecki, Claudia Nastasi, Thomas Litman, Anders Woetmann, Lise Mette Rahbek Gjerdrum, Niels Ødum

**Affiliations:** ^1^LEO Foundation Skin Immunology Research Center, Department of Immunology and Microbiology, University of Copenhagen, Copenhagen, Denmark; ^2^Department of Molecular Pharmacology, Zealand Pharma A/S, Glostrup, Denmark; ^3^PS Pharmaconsult, Allerød, Denmark; ^4^Center for Cancer Immune Therapy, Department of Hematology, Copenhagen University Hospital at Herlev, Copenhagen, Denmark; ^5^Department of Dermatology, Copenhagen University Hospital at Bispebjerg, Copenhagen, Denmark; ^6^Department of Pathology, Zealand University Hospital, Roskilde, Denmark

**Keywords:** Sézary syndrome, Kv1.3 channel, ShK, cutaneous T-cell lymphoma, cancer

## Abstract

The voltage-gated potassium channel Kv1.3 (KCNA3) is expressed by a subset of chronically activated memory T cells and plays an important role in their activation and proliferation. Here, we show that primary malignant T cells isolated from patients with Sézary syndrome (SS) express Kv1.3 and are sensitive to potent Kv1.3 inhibitors ShK and Vm24, but not sensitive to a less potent inhibitor [N17A/F32T]-AnTx. Kv1.3 blockade inhibits CD3/CD28-induced proliferation and IL-9 expression by SS cells in a concentration-dependent manner. In parallel, CD3/CD28-mediated CD25 induction is inhibited, whereas Kv1.3 blockade has no effect on apoptosis or cell death as judged by Annexin V and PI staining. In conclusion, we provide the first evidence that malignant T cells in SS express functional Kv1.3 channels and that Kv1.3 blockade inhibits activation-induced proliferation as well as cytokine and cytokine receptor expression in malignant T cells, suggesting that Kv1.3 is a potential target for therapy in SS.

## INTRODUCTION

Sézary syndrome (SS) is an aggressive leukemic form of cutaneous T-cell lymphoma (CTCL), which constitutes a heterogeneous group of extranodal non-Hodgkin lymphomas [1]. SS typically manifests as erythroderma and leukemic peripheral blood involvement by atypical malignant T cells named Sézary cells [2, 3]. SS patients experience heavy suffering from severe pruritus, desquamation and accompanying infectious complications [4]. The etiology is unknown, and genetic, epigenetic, and environmental factors have all been implicated in CTCL. Notably, a recent study on CTCL in a Danish cohort of twins showed no evidence for heredity playing a role in CTCL [5]. In parallel, an increasing body of evidence suggests that environmental factors may have both an etiological and a pathogenic role in CTCL [6–9]. Indeed, eradication of skin colonization by staphylococcus aureus through aggressive treatment with antibiotics is associated with a decrease in the fraction of malignant T cells in skin lesions [10] and staphylococcal alpha-toxin induces an increased ration between malignant and non-malignant T cells [11]. Importantly, the pathogenesis of CTCL including SS is closely associated with chronic inflammation and aberrant activation of nuclear factor-κB (NF-κB) pathway, nuclear factor of activated T cells (NFAT) pathway, and Janus kinase / signal transducer of activation (Jak/STAT) pathway in lesional skin and blood [12–15]. Inflammatory macrophages as well as dendritic cells are abundant in the CTCL tumor microenvironment [16, 17]. Recent genomic studies have reported activating mutations in genes such as PLCG1 promoting Ca^2+^/Calcineurin-NFAT signaling, leading to malignant proliferation in a fraction of CTCL patients [18]. However, the overall low recurrence of pathogenic small-scale mutations suggests that these are not the primary drivers of the disease. Notably, a recent study of single-cell heterogeneity showed a high degree of heterogeneity when comparing malignant T cells in different SS patients and even a considerable heterogeneity within the malignant T cell population in each individual patient [19, 20] suggesting that genetic instability may play a key role in CTCL. MicroRNAs are also important in the pathogenesis of CTCL, for some MicroRNAs (e.g., miR-21, miR-22, miR-155) have been reported to target cell-cycle regulators, signaling molecules and tumor suppressors, leading to enhanced proliferation, cytokine release, and resistance to apoptosis in malignant T cells [21–25]. Interestingly, loss of regulatory control through deletion, epigenetic silencing, or functional deviation of Jak/STAT signaling inhibitors such as SOCS1, SOCS3, SHP-1, and HNRNPK also appears to be implicated in aberrant cytokine signaling [26–30] indicating that a dynamic, multifactorial interplay between genetic, epigenetic and environmental triggers drive disease progression in SS and other variants of CTCL.

Current therapeutic strategies for SS involve multidisciplinary combinations of skin directed therapies and systemic chemotherapy, as well as immune-modulators (e.g., Interferon α, Bexarotene) and histone deacetylase inhibitors (HDACi, e.g., Vorinostat, Romidepsin). In recent years targeted therapies are emerging and a number of monoclonal antibodies have shown various efficacy in SS [31]. For instance, Mogamulizumab targeting C-C chemokine receptor 4 (CCR4) has been approved in 2018 for clinical treatment of SS [32, 33]. Alemtuzumab against CD52 gained considerable efficacy [34, 35], and Pembrolizumab against programmed cell death protein-1 (PD-1) showed activity among SS patients in clinical trials [36, 37]. Brentuximab vedotin as an antibody-drug conjugate targeting CD30 also demonstrated encouraging results in CTCL phase II/III trials [38, 39]. In addition, a newly developed oligonucleotide inhibitor of miR-155 named Cobomarsen is being evaluated in a phase II trial for CTCL [40]. However, despite all these therapeutic options, the prognosis of SS remains poor, and novel therapeutics are highly needed [4, 41].

The Kv1.3 ion channel was discovered on T lymphocytes over three decades ago [42]. It is a voltage-gated potassium channel residing in the plasma membrane that plays an important role in T cell activation by regulating membrane potential and calcium signaling. Kv1.3 channel-mediated K^+^ efflux maintains a negative membrane potential during T cell activation providing a driving force for calcium influx and a high concentration of cytoplasmic calcium (~ 1 μM) [43, 44]. The elevated cytosolic calcium following T cell receptor stimulation activates the phosphatase calcineurin that in turn dephosphorylates NFAT. Once dephosphorylated, NFAT translocates to the nucleus and initiates gene transcription, cytokine secretion, and T cell proliferation [45, 46]. Blockade of Kv1.3 influences Ca^2+^ homeostasis and inhibits T cell activation and proliferation. Kv1.3 channels have therefore been proposed to be a promising therapeutic target [45, 47]. In past years, Kv1.3 has been intensively investigated which has provided a series of encouraging results. For instance, in many autoimmune diseases, disease-associated autoreactive cells are found having high expression of Kv1.3 protein in the plasma membrane including multiple sclerosis [48], rheumatoid arthritis [49], psoriasis [50], and type-1 diabetes mellitus [49]. Moreover, selective Kv1.3 inhibitors have shown efficacy in preclinical animal models [48–50], and one inhibitor, Dalazatide, is now in clinical trials for psoriasis [51], suggesting that Kv1.3 may indeed be a promising therapeutic target in chronically activated T-cell disorders.

Malignant T cells in SS derive from chronically activated T memory cells [52]. Yet, it has to date not been investigated if malignant SS cells express Kv1.3. In this study, we explored the expression and possible functions of Kv1.3 in SS. We demonstrate that Kv1.3 is expressed on primary malignant cells of SS patients, and that Kv1.3 blockade inhibits malignant cell proliferation and cytokine signaling, without inducing apoptosis.

## RESULTS

### Kv1.3 is expressed by *ex vivo* isolated malignant cells from SS patients

**Figure 1:**
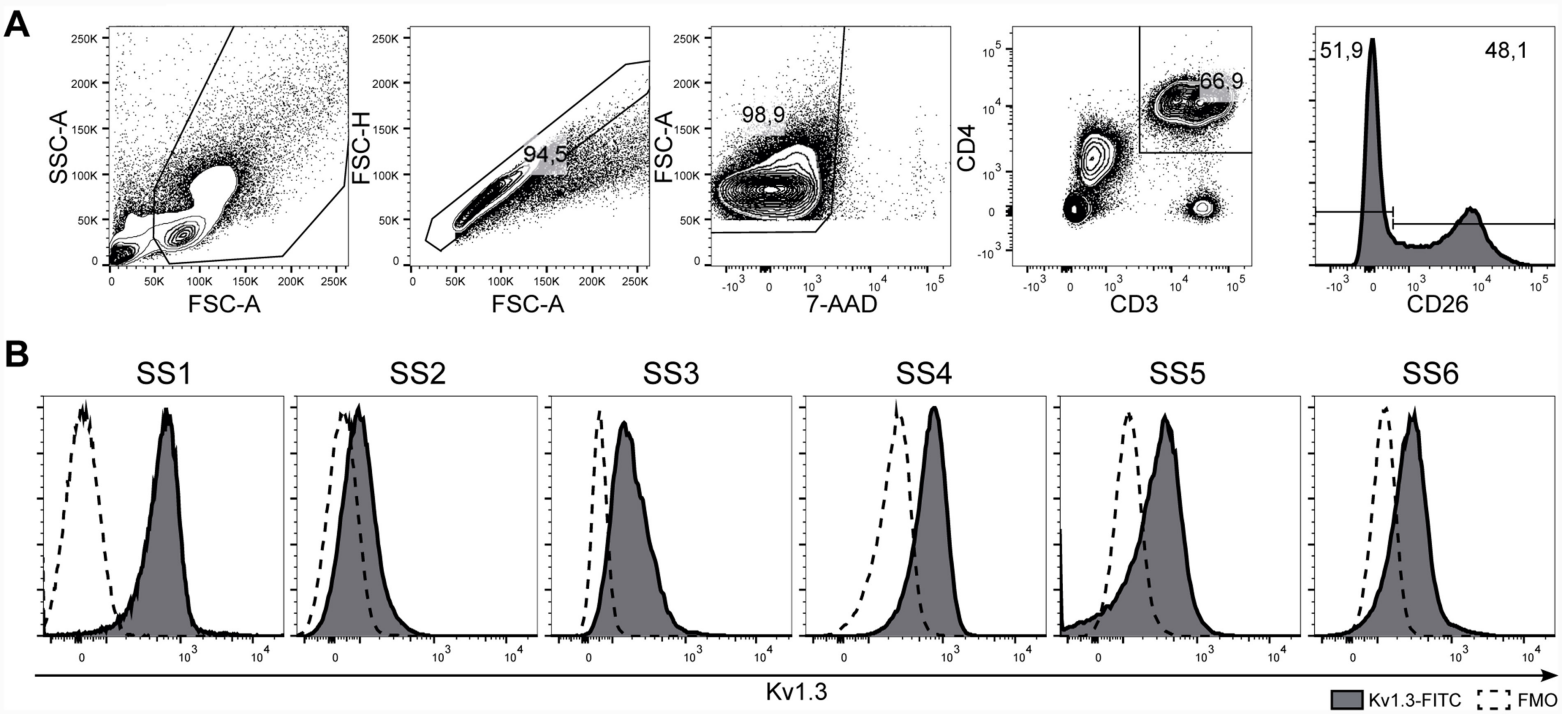
Kv1.3 is expressed in malignant cells from SS patients. PBMCs from six SS patients (SS1–SS6) were stained with anti-Kv1.3, CD3, CD4, CD26 antibodies, and 7-AAD and analyzed by flow cytometry. (**A**) Representative gating strategy for the identification of malignant T cells (CD3+CD4+CD26–). (**B**) Histograms of Kv1.3 protein expression on the malignant T cell population.

Kv1.3 has been reported to be highly expressed in many chronically activated T-cell diseases [48–50]. Yet, no studies have investigated the expression of Kv1.3 in SS. Accordingly we obtained peripheral blood mononuclear cells (PBMCs) from six SS patients and analyzed Kv1.3 expression using flow cytometry. Malignant cells were defined as CD26- within the CD3+CD4+ T cell population (Figure 1A). As shown in Figure 1B, Kv1.3 protein was expressed on the cell surface of malignant T cells from all six SS patients, although at varying levels.

### Kv1.3 blockade inhibits activation-induced proliferation of malignant T cells from SS patients

**Figure 2:**
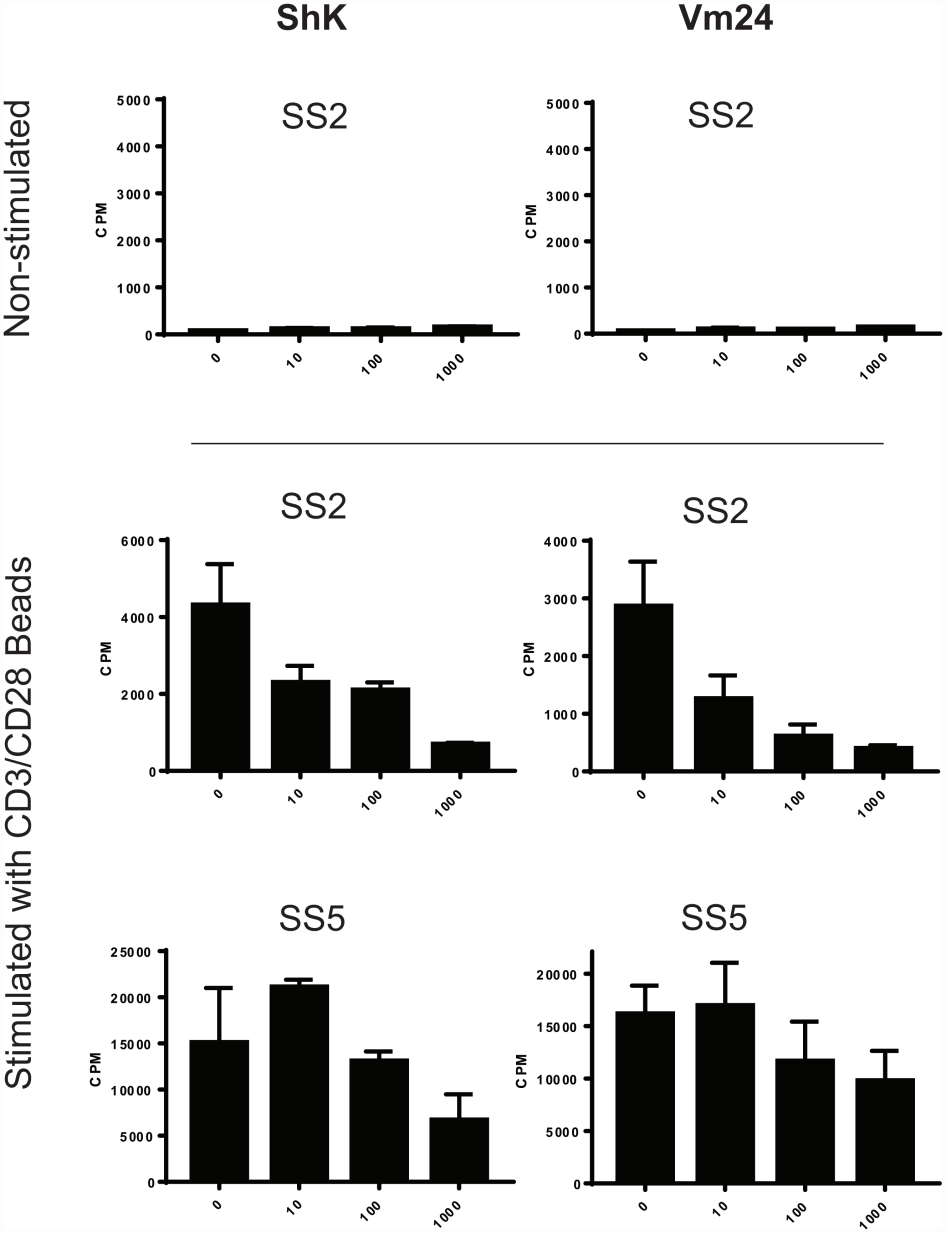
Kv1.3 inhibitors ShK and Vm24 inhibit activation-induced proliferation of PBMCs from SS patients. PBMCs from two SS patients were incubated with Kv1.3 inhibitors, ShK and Vm24, in 10-fold increasing concentrations (0, 10, 100 and 1000 nM) for 72 hours, with or without anti-CD3/CD28 beads. ^3^H-thymidine incorporation of the last 24 hours was measured as counts per minute (CPM). Data of two experiments with bead stimulation and one representative experiment without bead stimulation are shown. Error bars represent standard deviation (SD) of 3 technical replicates.

**Figure 3:**
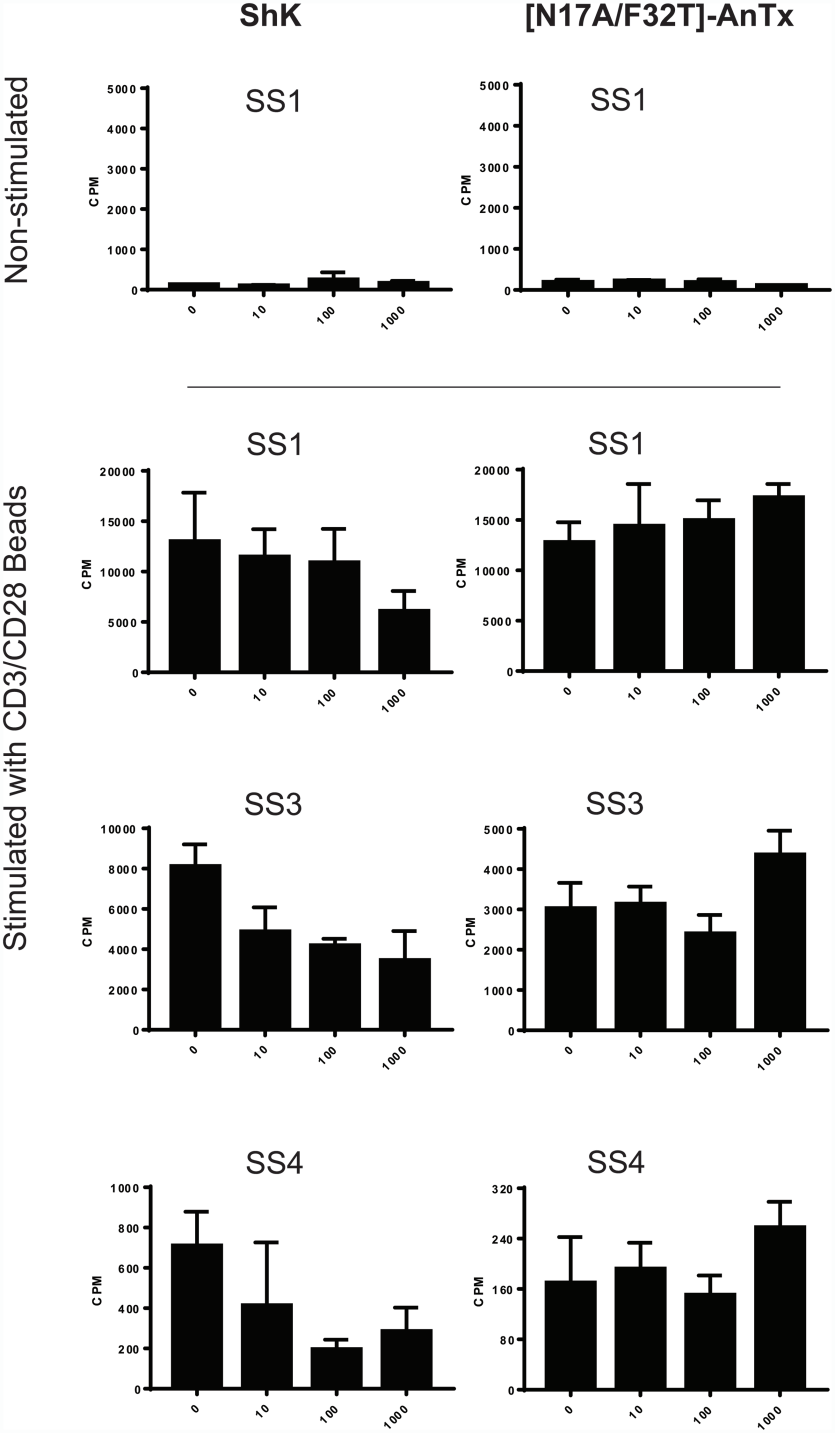
Kv1.3 inhibitor ShK inhibits activation-induced proliferation of PBMCs from SS patients, whereas [N17A/F32T]-AnTx shows little inhibition. PBMCs from three SS patients were incubated with Kv1.3 inhibitors, ShK and [N17A/F32T]-AnTx, in 10-fold increasing concentrations (0, 10, 100 and 1000 nM) for 72 hours, with or without anti-CD3/CD28 beads. ^3^H-thymidine incorporation of the last 24 hours was measured as counts per minute (CPM). Data of three experiments with bead stimulation and one representative experiment without bead stimulation are shown. Error bars represent standard deviation (SD) of 3 technical replicates.

Since Kv1.3 channels showed to be expressed by malignant T cells from SS patients, we next aimed to determine whether Kv1.3 augments cell proliferation. We used three well characterized Kv1.3 inhibitors with varied potency: two potent ones called ShK and Vm24, and a 150 times less potent one called [N17A/F32T]-AnTx, see Supplementary Table 1 [53–55]. We treated PBMCs from SS patients with 10-fold increasing concentrations of ShK (0, 10, 100 and 1000 nM), with or without anti-CD3/CD28 beads for 72 hours and measured the incorporation of ^3^H-thymidine during the last 24 hours of culture. While no ^3^H-thymidine incorporation was observed in the absence of bead stimulation, the ^3^H-thymidine incorporation of SS cells stimulated with anti-CD3/CD28 beads was inhibited in a dose-dependent manner by the potent Kv1.3 inhibitors, ShK and Vm24, suggesting that blockade of Kv1.3 inhibits the proliferation of SS cells (Figure 2). Likewise, ShK inhibited activation induced proliferation in another three patients (Figure 3, left), whereas [N17A/F32T]-AnTx had little effect (Figure 3).

**Figure 4:**
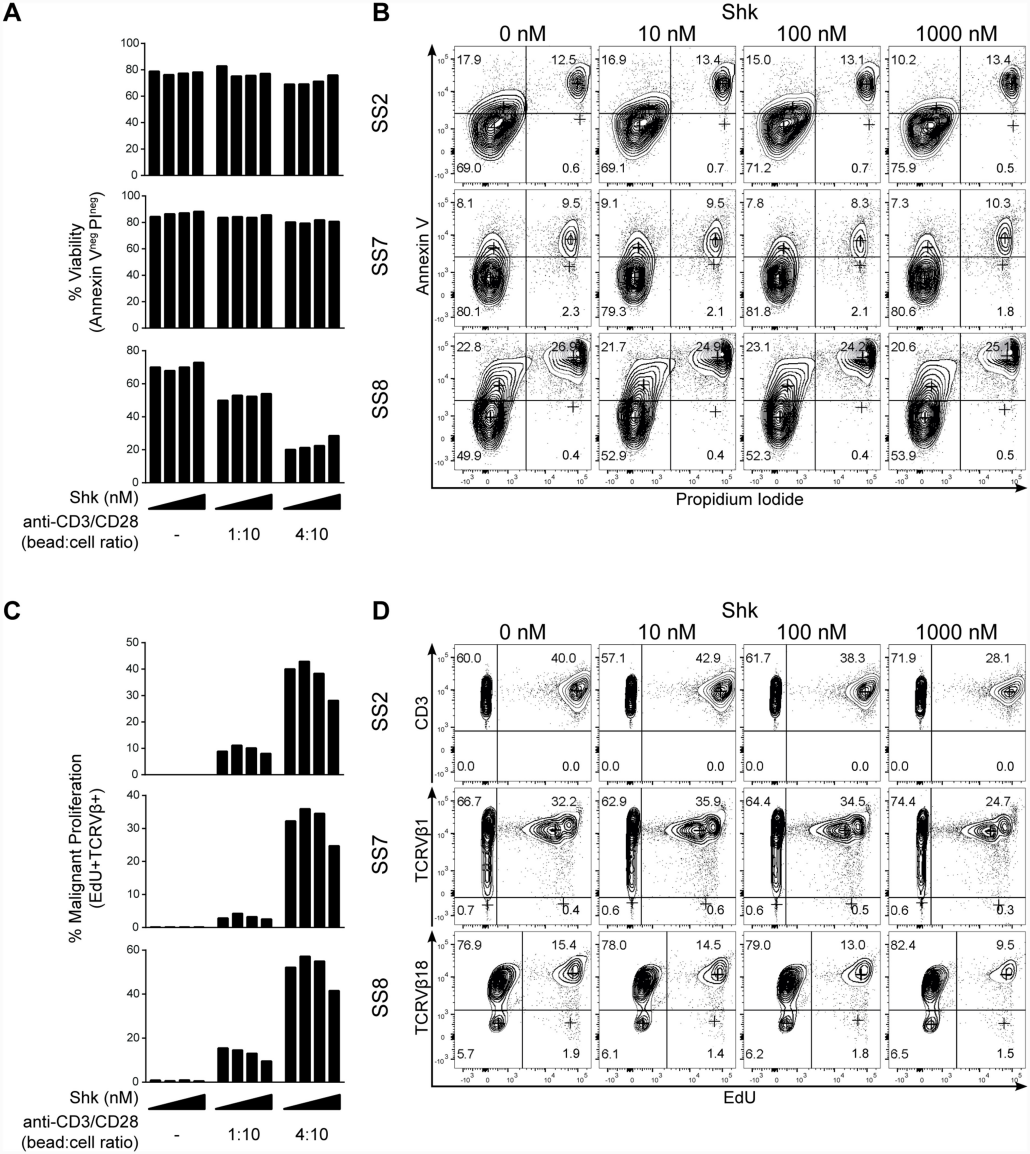
Kv1.3 blockade inhibits activation-induced proliferation of malignant T cells from SS patients. PBMCs from three SS patients were incubated with 10-fold increasing concentrations of ShK (0, 10, 100 and 1000 nM) for 72 hours in the presence or absence of anti-CD3/CD28 beads. EdU was added during the last 24 hours of culture. Anti-CD3, CD4, TCRVβ, EdU, Annexin V and PI were stained followed by flow cytometric analysis. Quantification of viability and representative flow cytometric plots are shown in (**A**) and (**B**). Quantification of malignant proliferation and representative flow cytometric plots are shown in (**C**) and (**D**).

To elucidate whether the reduction of ^3^H-thymidine incorporation in SS cells was caused by increased cell death, we analyzed the proportion of apoptotic and dead cells in response to ShK treatment by flow cytometry. PBMCs from three SS patients were treated with ShK and stained with Annexin V and propidium iodide (PI). As shown in Figure 4A and 4B, we observed high viability (Annexin V- and PI-) and low apoptosis (Annexin V+) in unstimulated malignant T cells from all three patients. Following anti-CD3/CD28 stimulation, some degree of activation-induced cell death (AICD) was observed in one patient (SS8) (Figure 4B, lower part), whereas little AICD was observed in the other two patients (Figure 4B, upper and middle part). The difference between the patients could be explained by the functional heterogeneity among SS patients [19]. Regardless, increasing ShK concentrations did not increase the proportion of apoptotic (Annexin V+) or dead (PI+) cells (Figure 4A and 4B), indicating that ShK-mediated blockade of Kv1.3 does not induce cell death. This finding is in line with previous reports indicating that some Kv1.3 inhibitors, including ShK, do not induce apoptosis [56, 57].

**Figure 5:**
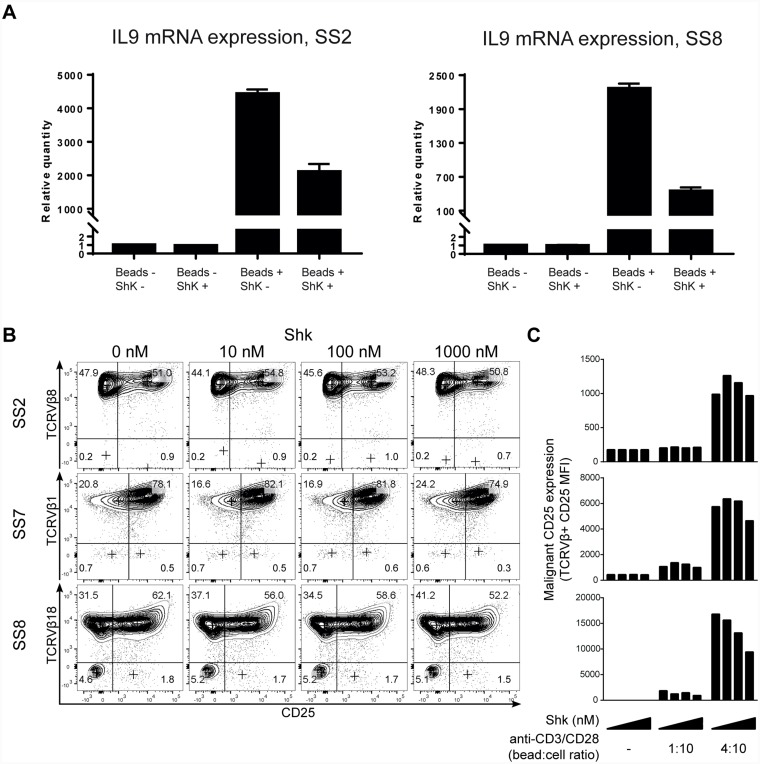
Kv1.3 blockade inhibits activation-induced cytokine and cytokine receptor expression in malignant T cells from SS patients. (**A**) PBMCs from two SS patients were treated with 1 µM ShK in the presence or absence of anti-CD3/CD28 beads for 72 hours. IL-9 mRNA expression was analyzed by quantitative PCR, with GAPDH as normalization control. Error bars represent standard deviation (SD) of 3 technical replicates. (**B** and **C**) PBMCs from three SS patients were incubated with different concentrations of ShK (0, 10, 100 and 1000 nM) for 72 hours in the presence or absence of anti-CD3/CD28 beads. Anti-CD3, CD4, CD25 and TCRVβ were stained followed by flow cytometric analysis. Representative plots of CD25 expression (B) and mean fluorescence intensity (MFI) of CD25 in malignant populations (C) are shown.

To confirm that ShK inhibited the proliferation of malignant T cells per se, we further analyzed the proliferative response at the single-cell level by measuring EdU (5-ethynyl-2′-deoxyuridine) incorporation using flow cytometry during the last 24 hours of 72 hour long ShK treatment. We found that, in the presence of anti-CD3/CD28 stimulation, the population of malignant T cells that had proliferated (marked by TCRVβ+EdU+) was decreased by ShK in a dose-dependent manner (Figure 4C and 4D). For patient SS2, CD3 was used as a surrogate of TCRVβ8 in the EdU staining and the feasibility was confirmed in a different staining showing that 99% of CD3+CD4+ cells were TCRVβ8+ (see Figure 5B). Collectively, these findings strongly suggest that Kv1.3 blockade inhibits the proliferation of malignant T cells in SS patients.

### Kv1.3 blockade inhibits activation-induced cytokine and cytokine receptor expression in malignant T cells from SS patients

As the T cell receptor (TCR)-mediated increase of intracellular free calcium is a key trigger of NFAT activation and expression of growth factors such as cytokines in healthy T cells [43, 44], we next addressed whether Kv1.3 blockade inhibited TCR-induced cytokine expression. Cytokines have been implicated in the pathogenesis of SS (reviewed in Ref. [15]) and recently, interleukin 9 (IL-9) was shown to promote T cell proliferation in healthy and malignant T cells [58]. To determine if Kv1.3 inhibition altered the cytokine expression in SS cells, we next determined the expression of IL-9 by quantitative PCR following treatment with or without 1 µM ShK in the presence or absence of anti-CD3/CD28 beads. As shown in Figure 5A, anti-CD3/CD28 stimulation strongly increased the mRNA expression of IL-9, compared to unstimulated cells. Notably, the CD3/CD28-mediated IL-9 induction was significantly inhibited by ShK, indicating that Kv1.3 plays an important role in TCR-mediated cytokine expression in SS T cells. This is consistent with our findings that ShK inhibits TCR-driven proliferation.

The high-affinity IL-2 receptor alpha chain (CD25, IL-2Rα) is a direct target of calcium activated NFAT in T cells and its aberrant expression has been reported in many lymphoid malignancies [59–61]. To determine if blocking Kv1.3 affected TCR-induced CD25 expression in malignant T cells, we examined CD25 expression on malignant T cells from three SS patients. Malignant T cells displayed pronounced CD25 expression following stimulation by anti-CD3/CD28 beads, which was partly inhibited by Kv1.3 blockade (Figure 5B and 5C). Thus, ShK induced a dose-dependent decrease of CD25 surface expression.

Taken together, we provide the first evidence that blocking Kv1.3 inhibits activation-induced expression of the cytokine receptor CD25, IL-9, and proliferation of malignant T cells.

## DISCUSSION

The present study provides the first evidence that Kv1.3 potassium channels are expressed and play a functional role in SS cells. Primary malignant T cells from investigated SS patients displayed a modest surface expression of Kv1.3. Blockade of Kv1.3 with the potent Kv1.3 inhibitors ShK and Vm24 inhibited TCR-mediated activation and proliferation of malignant T cells from SS patients. Notably, we observed some degree of heterogeneity in Kv1.3 expression among SS patients supporting recent reports on inter-individual heterogeneity in SS [19]. However, despite varied Kv1.3 expression, the TCR-induced proliferation of malignant T cells was suppressed by Kv1.3 blockade in all investigated patients. These findings show that Kv1.3 channels were not only expressed on the surface, but also functional in SS T cells. Interestingly, other studies have shown that malignant cells of B and T cell origin also express functional Kv1.3 channels [62, 63], implying that Kv1.3 expression and function is not lost during neoplastic transformation of B and T cells. On the contrary, Kv1.3 expression may provide a growth advantage to malignant cells. In support of this, Kv1.3 blockade inhibits activation-induced proliferation of malignant T cells (as shown in Figures 2–4), and inhibits cell proliferation in hematological cancers of B cell origin, as shown by others [62, 63].

It is well established that a key event in TCR-mediated activation of T cells involves the release of calcium from internal stores and an influx of extracellular calcium resulting in a steep increase in intracellular free calcium, which activates the protein phosphatase calcineurin to dephosphorylate and activate the transcription factor NFAT. In turn, this triggers nuclear translocation of NFAT and transcription of genes encoding cytokines and their receptors, eventually driving mitogenesis and T cell proliferation [43–46]. The mechanism by which Kv1.3 blockade inhibits cell proliferation has been elucidated in previous studies [44, 45, 48, 64]: Kv1.3 inhibitors including ShK block potassium efflux by attaching to and obstructing the central pore of Kv1.3 channel, which results in a reduced influx of extracellular calcium. Gradually, the reduced calcium influx decelerates calcineurin activation and NFAT signaling leading to a relative decrease in gene transcription, cytokine secretion and cell proliferation. In our study, we found that the Kv1.3 blocker ShK inhibited anti-CD3-CD28 bead-induced expression of the high-affinity IL-2 receptor CD25, transcription of the cytokine and malignant growth factor IL-9 [65], and T cell proliferation. Our data thus support the hypothesis that Kv1.3 channels are expressed and functional in malignant T cells.

While Kv1.3 inhibition has been reported to induce apoptosis in B-CLL cells [62], we did not observe increased apoptosis following Kv1.3 blockade in malignant T cells (Figure 4A and 4B), which is consistent with previous reports showing that ShK does not induce apoptosis in healthy T cells [57].

It is generally believed that SS and other variants of CTCL arise from chronically activated T cells as also reflected by their memory T cell phenotype [52]. However, it is unknown what drives this chronic stimulation of malignant T cells. Both endogenous and exogenous factors are believed to be involved. For instance, gain-of-function mutations affect signaling pathways to drive T cell activation, and the loss of regulatory control due to deletions in SOCS1 or epigenetic silencing of SHP-1, lower the activation threshold and may drive aberrant activation and proliferation [9, 26, 27, 29]. Exogenous factors such as bacteria and their toxins may also trigger or amplify chronic activation of malignant T cells [7]. Notably, malignant T cells induce structural changes in the skin, which compromises skin barrier function [66] and possibly paves the way for bacterial colonization of lesional skin by toxin producing Staphylococcus aureus, which is a characteristic feature of SS [67]. Moreover, Staphylococcal toxins induce activation of proto-oncogenes such as STAT3 and a release of inflammatory and immune regulatory cytokines [7]. Thus, it is likely that multiple factors – endogenous and exogenous – act in concert to induce proliferation of malignant T cells. In this context, Kv1.3 could be a potential target for therapy. Due to the moderate inhibitory effect of ShK on activation and proliferation of malignant T cells, Kv1.3 inhibitors may, at first glance, not appear as ideal therapeutic candidates for mono-therapy of SS. However, as SS patients notoriously develop drug resistance, it is feasible that Kv1.3 inhibition might have a role in combination-therapy together with other drugs targeting different disease mechanisms such as resistance to apoptosis. HDAC inhibitors (HDACi), such as vorinostat and romidepsin, induce apoptosis in SS cells [68, 69], but they may not be able to completely eliminate all malignant subpopulations in these patients [19], potentially leading to cancer recurrence and treatment resistance. Thus, it is likely that multiple drugs directed against different targets are needed to obtain high treatment efficacy in each individual patient. As such, Kv1.3 blockers might have a role in a personalized treatment approach to SS. Accordingly, studies are in progress to investigate the effect on primary SS cells *ex vivo* by ShK in combination with approved treatments such as HDACi, retinoids, and interferon-α.

In conclusion, the present study provides first evidence that malignant T cells from SS patients express functional Kv1.3 channels, and that Kv1.3 blockade inhibits activation induced CD25 and IL-9 expression and proliferation of SS cells, suggesting that Kv1.3 is a potential therapeutic target in SS.

## MATERIALS AND METHODS

### Patient cells

In accordance with the Declaration of Helsinki, the samples were obtained with written informed consent from all patients after approval by the Committee on Health Research Ethics. Peripheral blood mononuclear cells (PBMCs) were isolated from the blood of Sézary syndrome (SS) patients by density-gradient centrifugation, using LymphoPrep and Sepmate-50 tubes (Stem Cell Technologies, Cat# 07851 and Cat# 85460, Cambridge, UK). Malignant SS T cells typically lack the expression of cell surface marker CD26 and express a common TCRVβ chain [1]. Accordingly, T cells were identified as malignant (TCRVβ+ or CD26–) and non-malignant (TCRVβ- or CD26+).

### Kv1.3 inhibitors

ShK (a toxin from sea anemone Stichodactyla helianthus), Vm24 (a toxin from scorpion Vaejovis mexicanus smithi) and [N17A/F32T]-AnTx (a mutant toxin from scorpion Anuroctonus phaiodactylus) were synthesized and tested by Zealand Pharma A/S (Copenhagen, Denmark). A CHO cell line stably expressing human Kv1.3 channel (hKv1.3) was purchased from Perkin Elmer. hKv1.3 activities in different concentrations of Kv1.3 inhibitors were measured using the FluxOR Potassium Ion Channel Assay from Invitrogen as described by the manufacturer. The flux of thallium ions into the cells as a response to Kv1.3 activation was quantified using a Fluorometric Imaging Plate Reader (FLIPR^®^) instrument from Molecular Devices, shown in Supplementary Table 1.

### [Methyl-^3^H]-thymidine proliferation assay

Assays were performed in RPMI-1640 medium containing 2 mM L-glutamine, 0.5 units/mL penicillin, 0.1 mg/mL streptomycin (Sigma-Aldrich, Darmstadt, Germany) and 10% human serum (Bloodbank, Rigshospitalet, Denmark).

25,000 PBMCs were seeded per well in 96-well round bottom plates in different concentrations of Kv1.3 inhibitors, stimulated or non-stimulated with anti-CD3/CD28 beads (Cat# 11132D; Thermo Fisher) and incubated for 72 hours. ^3^H-thymidine (6.7 Ci; Perkin Elmer, Skovlunde, Denmark) was added 24 hours before cells were harvested onto glass fiber filter plates (Uni-Filter-96 GF/C; Perkin Elmer) using FilterMate cell harvester (Unifilter-96; Perkin Elmer). Filter plates were dried over night at room temperature, and scintillation fluid was added (MicroScint-O; Perkin Elmer), then ^3^H-thymidine incorporation was measured as counts per minute (CPM) in a scintillation counter (TopCount NXT; Parkard, Meriden, CT, USA).

### Flow cytometry

Antibodies against CD3, CD4, CD25, CD26, TCRVβ1, TCRVβ8 and TCRVβ18 as well as Annexin V were purchased from BD Biosciences, BioLegend, Miltenyi Biotec or Beckman Coulter. Dead cells were excluded using propidium iodide (PI). FITC conjugated Kv1.3 antibody was purchased from Sigma-Aldrich (Cat# P4247-50UL).

Single cell suspension was assured by filtering cells through a 100 µm cell strainer. For all stainings, antibodies were diluted in BD Brilliant Stain Buffer (Cat# 563794; BD Biosciences) and stained for 30 minutes at room temperature protected from light. For Annexin V staining, surface stained cells were resuspended in 50 µl Annexin V binding buffer (Cat# 556454; BD Biosciences) containing Annexin V and PI and incubated at room temperature for 15 minutes protected from light followed by addition of 150 µl Annexin V binding buffer and analyzed immediately. EdU (5-ethynyl-2′-deoxyuridine) incorporation was analyzed using the Click-iT^®^ Plus EdU Alexa Fluor^®^ 488 Flow Cytometry Assay Kit (Cat# C10632; Thermo Fisher) according to manufacturer’s instructions. PBMCs were seeded 100,000 cells per well in 96-well round-bottom plates in different concentrations of ShK, with or without anti-CD3/CD28 bead stimulation, and incubated for 72 hours. EdU was added to the wells 24 hours before cells were harvested.

Flow cytometric analysis was conducted on a 5 laser BD LSR-Fortessa at the Core Facility for Flow Cytometry at the University of Copenhagen. Data were analyzed using the FlowJo software (Treestar, Ashland, OR, USA).

### RNA purification and quantitative PCR

Total RNA was purified with miRNeasy Mini Kit (Cat# 217004; Qiagen), and cDNA was transcribed from 450 ng RNA using High-Capacity cDNA Reverse Transcription Kit (Cat# 4368813; Applied Biosystems). All samples were DNAse treated before cDNA synthesis using DNase 1 (Cat# AMPD1-1KT; Sigma-Aldrich). Quantitative PCR (qPCR) was performed using Taqman Gene Expression assay probes/primers (Thermo Fisher) and LightCycler 480 probes master mix. Amplification was performed using a LightCycler 480 II (Roche, Hvidovre, Denmark). mRNA expression is presented as relative quantity to the control determined by ddCt method. IL-9 (Cat# Hs00174125-m1) and GAPDH (Cat# Hs02786624-g1) probes were purchased from Thermo Fisher.

## SUPPLEMENTARY MATERIALS


