# Supplementary material for: Expression and function of Kv1.3 channel in malignant T cells in Sézary syndrome
Source: Oncotarget. 2019 Aug 6;10(47):4894–906. doi: 10.18632/oncotarget.27122 (PMC6690676; doi:10.18632/oncotarget.27122)
Supplement: Supplementary file 1 [file oncotarget-10-4894-s001.pdf]

Expression and function of Kv1.3 channel in malignant T cells in Sézary syndrome

SUPPLEMENTARY MATERIALS

Supplementary Table 1: Potency of three Kv1.3 inhibitors

Description	hKv1.3 FLIPR* (nM)	Origin	Species of toxin
ShK	0.1	Sea anemone	Stichodactyla helianthus
Vm24	0.07	Scorpion	Vaejovis mexicanus smithi
[N17A/F32T]-AnTx	15	Scorpion	Anuroctonus phaiodactylus

*Fluorometric Imaging Plate Reader (FLIPR®).
